# Pathological Importance of Ulceration of Os Uteri

**Published:** 1854-11

**Authors:** Charles West

**Affiliations:** Fellow of the Royal Royal College of Physicians; Physician Accoucher to St. Bartholomew’s Hospital; and Physician to the Hospital for Sick Children, London, 1854


### AN ENQUIRY INTO THE PATHOLOGICAL IMPORTANCE OF ULCERATION OF THE OS UTERI. BEING THE CROONIAN LECTURES FOR THE YEAR 1854.

BY CHARLES WEST, M. D.,

Fellow of the Royal Royal College of Physicians; Physician Accoucher to St. Bartholomew's Hospital; and Physician to the Hospital for Sick Children,
London, 1854. 8vo. pp. 95.

It is time that an accurate and unbiased investigation should be made into the present conddition of our knowledge as respects the diseases of the uterus, and their proper treatment; to determine how far the opinions promulgated in regard to uterine pathology are based on correct observation, and the remedial measures deduced from these opinions are calculated to fulfil the necessary indications.

For a long period the several morbid conditions of the uterus were either entirely overlooked or their frequency and importance underrated, and, as a necessary consequence, their therapeutal management was irrational, inefficient, and empirical. Of late years, however, an impulse in an opposite direction has been given to uterine pathology. Various morbid conditions of the uterus, but more especially of its os and cervix, are now considered by many physicians, not only to be of extreme frequency, but the cause of many of the derangements of the other organs, as well as of the various general disturbances of health, to which the female is liable. So far, indeed, is this metritic pathology carried by a few, that, in the investigation of the diseases of the female sex, they would seem to view the speculum uteri as of equal, if not more importance, than is the stethoscope in the exploration of the morbid conditions of the heart and respiratory organs. In too many instances, it is to be feared that they have inverted the pathological series, and, in directing, too exclusively, to certain abnormal conditions of the neck and orifice of the uterus, that have been brought to light since the introduction of the speculum, they have placed cause for effect, and effect for cause, while many important questions in reference to the diseased conditions of the uterus, their true character, causes, and results, have been allowed to pass without even an attempt at investigation. Nor does the evil stop here. The exclusiveness of the views referred to reacts injuriously upon him who entertain them. As Dr. West very truly remarks—

"He unlearns what physiology might teach him of the uterus and its functions, and sees in all the varied manifestations of disorder the expression of one fact, and one fact alone; namely, the existence of ulceration at the orifice of the womb, and its reaction first on the uterine system, then the general health. For him, indeed, there is little more to learn in uterine pathology; for when once a case has been ascertained not to be one of fibrous tumour, polypus, or cancer, then ulceration of the os uteri is the almost invariable cause to which the symptoms are referred, and the cure of this ulceration is the one grand object at which he endeavors. All the evils inseparable from the practice of a speciality are thus aggravated, and the natural tendency of such practice to subside into routine, or to degenerate into empiricism (I use the word in no invidious sense) becomes almost unavoidable."

The frequency with which the uterus becomes the seat of disease, and the importance of all its morbid conditions in their influence upon the comfort, health, and, occasionally, upon the life of the female, are such as to demand from the medical practitioner, the closest investigation, that he may become acquainted with their nature, their causes, and distinctive characters, and the means best adapted for their prevention and cure.

In the lectures before us, Dr. West presents the results of a very logical and apparently candid inquiry into the pathological importance of ulceration of the os uteri; a question of deep interest at the present day, in consequence of the disputes to which it has given rise. Although the conclusions to which the lecturer has arrived are in direct opposition to the opinions of those who maintain that inflammation of the cervix and ulceration of the os uteri are the first and the last links in the chain of uterine pathology, they nevertheless claim the serious consideration of the medical practitioner.

The important question discussed in these lectures is—

"Whether ulceration of the os uteri is to be regarded as the first in a train of processes which are the direct or indirect occasion of by far the greater number of the ailments of the generative system; or whether, on the other hand, it is to be considered as a condition of slight pathological importance, and of small semeiological value —a casual concomitant, perhaps, of many disorders of the womb, but of itself giving rise to few symptoms, and rarely calling for special treatment."

Dr. West arranges the evidence by which he has endeavored to solve the question just stated under four principal heads:—

Under the *first* head is included the evidence deduced from what we know of the anatomy and physiology of the uterus in a state of health. The evidence derived from this source, Dr. West, however, admits, cannot from its very nature, be conclusive.

"It may render a certain occurrence probable or improbable, may substantiate or disprove the correctness of certain opinions or explanations, but cannot invalidate the evidence of positive facts."

Under the *secand* head is included the evidence derived from morbid anatomy. Whether examination of the dead body shows the morbid conditions of the os uteri which have been described to be frequent or rare, slight or extensive ; and whether we can make out what connection subsists between ulceration of the os and cervix uteri, and other changes in the tissues of the organ.

"It must, however, be borne in mind," remarks Dr. W., "that many evidences of disease, such as are very obvious during life, may be greatly obscured, or may even entirely disappear after death: and further, that uterine disorders of the class which we are considering, though exceedingly painful, and seriously interfering with a woman's health and comfort, are yet not of a kind to prove the direct occasion of her death. Evidence derived from this source will therefore be open to the objection that it understates both the frequency and the importance of these diseases."

Under the *third* head is adduced the evidence derived from the characters, course, and consequence of ulceration of the os uteri, as it presents itself to our notice, unconnected with other disease, in the case of the procident uterus.

"But," observes Dr. W., "whatever conclusions we may deduce from this source are open to all the objections inseparable from analogical reasoning. The probabilities of certain occurrences taking place in the uterus under other circumstances may be increased or weakened, but the evidence still falls short of absolute proof, either of the affirmative or of the negative character."

Under the *fourth* head is comprised the evidence derived from clinical observation. The determination from clinical experience, as to the frequency of ulcerations of the os uteri under those circumstances in which they ordinarily come under our notice, and call, or are supposed to call, for our interference, as well as the conditions generally associated with the ulceration, and the symptoms to which it commonly gives rise.

"If," Dr. W. remarks, "the alleged symptoms of ulceration are found to be not rarely present without ulceration, and if ulceration is undiscovered even where there are no symptoms; or if, in the the same case, the ulceration may vary in extent, with no corresponding change in the symptoms; if if an indurated state of the cervix uteri exists without ulceration, and ulceration even of long standing, without induration—the conclusion, especially if supported by the answers obtained to our previous inquiries, seems to me irresistible, that the importance of inflammation of the cervix, and of ulceration of the os uteri, has been overstated; that they are not the cause of all the symptoms which they have been alleged to occasion, and that, in the treatment of uterine disease, many other considerations must influence us more than the mere removal of ulceration of the orifice of the womb."

In regard to the testimony derived from anatomy and physiology, the lecturer shows, that in organization and physiological importance the cervix is inferior to the body of the uterus; that it is much less liable to morbid alterations in its intimate structure, and sustains, with surprising impunity, mechanical violence and the contact of the strongest caustics.

"But," he remarks, "if structurally so lowly organized—if physiologically of such secondary importance—if so much less subject than the body of the uterus to alterations in its intimate structure and if so comparatively insensible even to rude modes of therapeutical interference—it certainly does appear to me that the assumption that some slight abrasion of the mucous membrane covering this part is capable of causing a list of ills so formidable as are attributed to it, ought to rest for its support upon some other and stronger foundation than any inference fairly deducible from anatomical or physiological data.

Under the second head of evidence, Dr. W. presents the results of the examination of *sixty-two* uteri taken from patients who died in the medical wards of St. Bartholomew's Hospital of other than uterine disease.

"Of the total number, 13 were above forty-five years of age, the remaining 49, between the years of fifteen and forty-five. Concerning all of the former class, and 30 of the latter, making a total of 43, it was either known with certainty, or concluded with great probability, that they were married, or had had sexual intercourse; the remaining 19 were believed to be virgins.

"The subjoined table shows the general results of the examination of the uterus in these cases, and the relations borne to ulceration of the os uteri by the more important morbid appearances.

| | | |
|---|---|---|
| Uterus healthy in . . . . . . | | 33 |
| " diseased in . . . . . . | | 29 |
| Ulceration of os uteri in . . . . . | | 17 |
| Ulceration existed alone in . . . . | 11 | |
| " with diseased lining of uterus in | 3 | |
| " with induration of walls of uterus in | 3 | |
| | — | 17 |
| Induration of walls of uterus, without ulceration of os | | 5 |
| Disease of lining of uterus, without ulceration of os | | 7 |
| Total of diseased uteri, . . . | | 29 |

"The os uteri was abraded in one of the subjects above forty-five years of age, and the lining of its interior was diseased in five of that number. In eleven of the nineteen patients, all under forty-five years old, who were virgins, the uterus of was perfectly healthy; in eight, it presented some sign or other of disease. This consisted five times in slight abrasion of the os uteri, which existed alone in three cases, but was associated in the other two with some morbid state of the interior of the womb. Twice the interior of the uterus was the only part affected, and once the uterine walls were much harder than natural.

"There is certainly something," remarks Dr. W., "at first not a little startling in the result at which we arrive, that the womb was found in a perfectly healthy condition in little more than the half of sixty-two women, none of whom died of uterine disease, nor were supposed to be suffering from any grave uterine ailment. But it, it ought indeed, to be asked, what is the value of these appearances? Some of them may be of little moment, and the very frequency of their occurrence, instead of substantiating the opinion that they are of great importance, rather militates against that supposition. When ulceration of the os uteri was first observed it was natural enough to attribute to it many symptoms, and to refer to its influence many structural changes. But what if such ulceration be found to be usually very limited in extent, and so superficial as to be unassociated with changes in the basement membrane of the affected surface, and exercising so little influence on the state of the uterus in general as to be unconnected, in a large number of instances, with changes either in the interior of the womb or in its substance, while induration of the uterine tissue and disease of the lining membrane of the womb are found independent of it or of each other? Should such appear to be the case, it will, I think, be rendered in the highest degree probable that this abrasion of the os uteri has not the long train of sequences which have been supposed to follow it, but that it is of comparatively small pathological import; that it may be found to vary under the influence of comparatively trifling causes; and not unfrequently to be dependent up on functional disorder of the uterus, just as the mucous membrane of the tongue and mouth betrays the disturbance of the digestive system; that it may, in short, be the consequence and sometimes the index, but rarely the occasion of the ailments with which it associated.

"Abrasion of the os uteri was observed in eleven instances unconnected with any other morbid condition of the womb. In six cases it was extremely slight, affecting just the edges of the os uteri, but not extending for more than a line in breadth; the mucous membrane lining the canal of the cervix was in all of these instances quite pale, but twice the lining of the uterine cavity was of a brighter red than natural. In the other five cases, the abrasion, though retaining the same character, was more extensive; once the abraded surface presented a finely granular aspect, but was quite uniform; but in the other four cases it had an uneven worm-eaten appearance, probably due to a partial destruction of the papillæ which beset the os uteri. In four of these cases the abrasion extended for a short distance up the canal of the cervix, while once it was limited to that exclusively, the lips of the os being perfectly pale and healthy, and the mucous membrane of the cervix unaltered, excepting along a strip of a third of an inch in breadth by an inch in length, where the posterior wall was abraded. In three of the above four instances there was some increase of vascularity in the mucous membrane of the cervix, which on one occasion extended for nearly half an inch up its canal; and once this condition was very marked, and the mucous membrane appeared swollen and infiltrated, but in no other case was there any appearance of thickening of the membrane either at the seat or in the immediate neighborhood of the abrasion.

"Under what circumstances is induration of the uterine tissue met with, and in connection with what other changes in the organ? It existed in nine cases: in five of which it was not associated with any other disease of the uterine substance; in three, it coexisted with ulceration of the os; and in one, with a morbid state of the interior of the uterus. In an unmarried girl, aged eighteen, who died of cardiac dropsy, the tissue of the fundus, and of the upper half of the body of the uterus, presented its usual characters; but about half-way down the body of the organ there began a strip of a dead yellow color of much denser texture, resembling fibro-cartilage or the elastic coat of an artery. The dense tissue lay immediately beneath the lining membrane of the uterus, and being at first only one line in thickness, increased in width till it came to constitute the whole thickness of the cervix uteri. In the case of another patient, aged forty-seven, a similar condition was met with in the body of the uterus, but scarcely at all involved the cervix: and in three other cases, in all of which the women were under thirty years old, the cervix uteri alone was affected, being white, hard, creaking under the knife, and seeming, under the microscope, to be composed of an extremely dense fibrous tissue.

"It appears, then, that most marked induration of the tissue of the cervix, and of part of the body of the womb, may exist where there is no other trace of inflammation either past or present. It may also occur in connection with inflammation and ulceration of the lining membrane of the uterine cavity. In a woman, who died at the age of fifty-six, about a third of the thickness of the wall both of the body and neck of the womb was exceedingly firm and creaked under the knife. Abundant glairy secretion from the cervical glands, and some want of transparency of its lining membrane, were the only unusual conditions of the interior of the uterine neck; but the cavity of the organ contained a copious purulent secretion mixed with blood; its mucous membrane was thickened, vascular, and destitute of polish, and about the middle of the posterior wall comepletely destroyed, leaving the substance of the womb beneath uneven, rather soft, and presenting the appearance of a granulating surface.

"Ulceration of the os uteri, and induration of the uterine walls, were associated together in three instances. On one occasion the ulceration was but slight, and the interior of the cervix extremely pale, though there was great injection of the lining of the uterine cavity. In this instance the cervical wall was much indurated, that of the body of the uterus rather less so. Extreme induration of the cervix existed in one case where there was rather extensive ulceration of the os uteri; and, in this instance, the cervix was considerably hypertrophied. The patient from whom this uterus was taken had been under my care for some years previously, suffering from symptoms such as Gooch describes under the name of irritable uterus; her sufferings had been most severe, and the enlargement of her womb most considerable at a time when there was no abrasion of its orifice. In one case only, in which there was considerable induration of the cervix, there was a distinct line of congestion about half a line in depth, between the ulcerated surface and the pale tissue of the undurated cervix.

"In ten cases, the condition of the lining membrane of the uterine covity deviated from that which characterizes it in a state of health. Thrice this state of the interior of the womb coexisted with ulceration of its orifice of moderate extent, and presenting its ordinary appearance; but in the remaining seven instances the os uteri was perfectly healthy. In seven of the ten cases the uterine mucous membrane was vividly injected so as to present a bright rose tint, and was more or less swollen and softened. Once very extensive disease of the lining membrane of the uterine cavity, probably of a tuberculous character, was discovered in the body of a woman fifty-six years old. In a second case, in which the patient was stated to have had a copious leucorrhœal discharge, and to have complained of pain and of a sense of heat at the lower part of the abdomen, the intensely red mucous membrane of the uterine cavity presented an almost gelatinous appearance, and looked not unlike decidua. In this instance, though there was some ulceration of the os, yet the lining membrane of the cervix was quite pale; no secretion occupied its canal, and the tissue of the uterus was quite healthy. In a third case, a small patch of ecchymosis was present beneath the lining of the uterine cavity; and in a fourth, where the patient had not menstruated for five months, the lining membrane, though of a pinkish color, had lost its polish, and looked more like an injected serous membrane than like the mucous lining of the womb."

In relation to many of the above abnormal appearances, they ought probably, Dr. W. believes, to be classed with pseudo-morbid rather than with pathological conditions; but the data, he remarks, at present fail us for distinguishing with accuracy the one from the other.

"But, be this as it may, it is yet abundantly evident that many of them imply deviations from a healthy state more considerable than the trifling abrasion or ulceration of the os uteri, which existed on several occasions. We have seen that, in by far the majority of cases, the ulceration, when present, was not merely trifling in extent, but that it had not given rise to so much irritation of the neighboring tissues as to produce any appreciable congestion of the mucous membrane in its vicinity, while the changes in the uterine substance alleged to depend upon it were oftener present without than in connection with it; and, moreover, none of the alterations about the os and cervix of the womb were so considerable as those which were apparent in its cavity."

Under the third head of evidence, Dr. W. examines the effectscommonly produced by ulceration of the os uteri in cases of prolapse of the womb beyond the external parts, and the symptoms to which it generally gives rise, and where we can trace the ulceration in its progress; can watch it for weeks or months together, and even see what it has led to when it has existed for years.

"It can scarcely be necessary to say," remarks Dr. W. in summing up his conclusions under this head of the inquiry, "that it is not my intention for one moment to assert that misplacement of the womb produces no inconvenience, or that ulceration of its orifice, when it is thus misplaced, is of no importance. Daily experience yields abundant proof to the contrary; but a detail of the symptoms or prolapse of the uterus forms no part of our present object. I referred to the accident and its consequences only for the sake of suggesting the resonable inference that, if inflammation of the neck of the womb were as frequent as has been supposed, or ulceration of its orifice the necessary occasion of such serious disorder of function and alteration of structure, we ought to meet with some of the most striking illustrations of these facts in cases where the womb, by its misplacement, is exposed to injuries from without, such as it was never intended to encounter.

"But though it be conceded, as I think it must be by all observers, that the symptoms supposed to characterize inflammation of the neck of the womb, and ulceration of its orifice, are not met with either constantly or in a specially marked degree in cases of prolapsus or procidentia uteri; still, we should not be justified in drawing an absolute conclusion from what we observe in the misplaced uterus, as to the effects produced by similar ailments attacking the organ when in its natural position. It may be alleged, and with plausibility, that during the gradual process of its displacement, the sympathies of the womb have been rendered less keen than they were while the organ retained its natural position; and that thus it comes to bear, with comparative impunity, injuries which might otherwise have produced great disorder of its functions and great alteration of its tissue."

"Under the fourth and last head of the inquiry: What clinical observation generally teaches us concerning ulceration of the os uteri, its course, its symptoms, and its importance? the question to which especial attention is directed by the lecturer are, whether sterility is more frequent, whether the rate of fecundity is lower, and whether abortion occurs oftener in cases marked by the presence of ulceration of the os uteri than in those in which ulceration is not present? Whether menstrual disorder is more common, more severe, or different in kind; whether leucorrhœa is more abundant, or furnished from a different source; or whether pain is less tolerable in the one class of cases than in the other. And, lastly, whether similar or different causes produce the uterine affections in the two classes of cases; whether the duration of illness is the same, and whether the structural alterations of the womb are alike or diverse?

The materials from which the lecturer has endeavored to make some approach to a satisfactory answer to these questions are derived from 1226 cases, of which records were preserved while the patients were under his care, either at the Middlesex or at St. Bartholomew's Hospital. Of these, 300 were in patients of one or other institution, and the remaining 926 were out-patients of St. Bartholomew's Hospital, between January 1, 1850, and October 15, 1853.

The investigation of the materials thus derived, as presented by the lecturer; the analysis and collation of the facts they present in their bearing upon the questions proposed for investigation, are valuable and interesting; they are well deserving of a careful and candid examination. We shall not pretend to present an abstract of them. To form a just estimate of their accuracy and the correctness of the author's deductions from them, they must be studied in detail. We can only lay before our readers the general conclusions to which the author has been led.

"1 Uterine pain, menstrual disorder, and leucorrhœal discharges —the symptoms ordinarily attributed to ulceration of the os uteri— are met with independently of that condition almost as often as in connection with it.

"2. These symptoms are observed in both classes of cases with a vastly predominating frequency at the time of the greatest vigor of the sexual functions, and no cause has so great a share of their production as the different incidents connected with the active exercise of the reproductive powers. But it does not appear that ulceration of the os uteri exerts any special influence, either in causing sterility or in producing abortion.

"3. While the symptoms are identical in character in the two classes of cases, they seem to present a slightly increased degree of intensity in those instances in which ulceration of the os uteri existed.

"4. In as far as could be ascertained by careful examination, four-fifths of the cases of either class presented appreciable changes in the condition of the uterus—such as misplacement, enlargement, and hardening of its tissues, while frequently several of these conditions coexisted. An indurated or hypertrophied state of the cervix uteri was, however, more frequent in connection with ulceration of the os uteri than independently of that condition.

"5. The inference, however, to which the last-mentioned fact would seem to lead, as to the existence of some necessary relation —such as that of cause and effect—between ulceration of the os uteri and induration of its cervix, is in great measure negatived by two circumstances:—

"1. The number of instances in which an indurated cervix coexisted with a healthy os uteri.

"2. The fact that, while induration of the cervix was present in 25 out of 46 cases in which the ulceration of the os was very slight, it was altogether absent in 9 out of 16 cases in which the ulceration was noted as having been very extensive."

Thus, it will be perceived that the final conclusion of Dr. West's inquiry into the pathological importance of ulceration of the os uteri is, that such ulceration is neither the general cause of the symptoms which have been attributed to it, nor even a general concomitant of them, and an index of their degree and severity.

In the third and concluding lecture, Dr. W. enters into a brief inquiry into the actual causes of the various morbid phenomena, that are considered by many as dependent solely upon ulceration of the os uteri.

These causes he believes to be very various, sometimes independent of local disease, as in the case of chlorosis, of hepatic disorder, of granular disease of the kidneys, of the gouty or rheumatic patient, all of which instances illustrate the dependence of uterine disorder on constitutional disease. Ulceration of the os uteri, when it occurs in such cases, Dr. W. considers to be of secondary importance, and equally so in many instances where disease really begins in the uterus itself, as in ailments succeeding to pregnancy, abortion, delivery, etc. In these latter cases, Dr. W. believes that the interior of the womb as well as in other instances where the symptoms of sexual disorder have succeeded to marriage, or where they have followed suppressed menstruation, and in those also in which uterine misplacements are succeeded by signs ef sexual disorder, or where these signs have been associated with misplacement of the ovary.

Referring to the treatment pursued by those who advocate the pathological importance of ulceration of the os uteri, Dr. W. remarks :—

"It may be asked, how is it that such successful results have followed a course of treatment directed exclusively to the cure of the ulceration—that the application of caustic to the os uteri has been succeeded by the restoration of the patient to health? Now, I think, it should be borne in mind that, in connection with this mode of treatment, various other measures are of necessity adopted, eminently calculated to relieve many of the slighter forms of uterine ailment. The married woman is for a time taken from her husband's bed, the severe exertion to which either a sense of duty urged, or a love of pleasure prompted her, is discontinued; while rest in the recumbent posture places the uterus and the pelvic viscera in just that position in which the return of blood from them encounters the smallest difficulties. The condition of the bowels, probably, before habitually neglected, is now carefully regulated, and the patient's diet, bland, nutritious, and unstimulating, often differs widely from that with which, while all her functions are over-taxed, she vainly strove to tempt her failing appetite. Add to this, that the occurrence of the menstrual period is carefully watched for; that all precautions are then redoubled, and each symptom of disorder, such as, on former occasions, had been borne uncomplainingly, though often not without much suffering, is at once encountered by its appropriate remedy; while, generally, returning convalescence is met in the higher classes of society by a quiet visit to the country, or to some watering-place, in pursuit not of gayety but of health; and we have assembled just those conditions best fitted to remove three out of four of the disorders to which the sexual system of woman is subject. But the very simplicity of these measures is a bar to their adoption; for you will bear me out in saying that the rules which common sense cannot but approve, but which seem to require nothing more than common sense to suggest them, are just those to which our patients least readily submit. The case is altered, however, when these said rules are laid down not as the means of cure themselves, but only as conditions indispensable to the success of that cauterization which, repeated once or oftener in the week, is the great remedy for the ulceration that the doctor has discovered, and which he assures his patient, and with the most perfect good faith, produces all the symptoms from which she suffers. The caustic used in these milder cases is the nitrate of silver; the surface to which it is applied is covered by a thin layer of albuminous secretion, which it is not easy to remove completely, and which serves greatly to diminish the power of the agent, while the slightly stimulating action that it nevertheless exerts seldom does harm; sometimes, I believe, does real good, though no more than might have been equally attained by vaginal injections, or other similar remedies, which the patient might have employed without the intervention of her medical attendant."

Notwithstanding it is the conviction of Dr. West, that in the great majority of instances in which the nitrate of silver is applied to the os uteri, the proceeding is simply superfluous, while to the use of caustic potash in cases in which the neck of the womb is more or less enlarged, he is altogether adverse, still, he admits that there are some exceptional cases in which ulceration or some allied morbid condition of the os uteri is found to exist, independent of any appreciable disease elsewhere; and others, equally rare, in which, after symptons of uterine ailment have been subdued, a morbid state of the os uteri persists, that are benefitted by stimulant applications.

"In such cases," he observes, "I use either the nitrate of silver, or the acid nitrate of mercury, though neither of them frequently; and for weeks together no case appears among my patients at St. Bartholomew's Hospital, in which the employment of either appears to me indicated. In justice to others, it should, I think, be observed, that we have no right to infer that the majority of practitioners who resort to these agents with much greater frequency than some of us feel warranted in doing, regard them as absolutely the best remedies that could be used, but merely as the best under the peculiar circumstances in which uterine diseases have to be treated.—Were it possible to keep any of those milder agents in contact with the abraded os uteri which can generally be applied to an irritated or ulcerated surfaces elsewhere, this would doubtless be allowed, in many instances, to be a preferable proceeding. The problem, however, is to find some agent sufficiently powerful to exert an influence which may continue for several days, and thus to obviate the necessity for that frequent painful interference which would otherwise be required. That lotions, baths, and other remedial agents, which may be safely entrusted to the patient herself, will answer the desired ends more frequently than some practitioners imagine, is my firm conviction, but I could not refrain from stating what seems to me to be the candid interpretation of their conduct who pursue a different course of proceeding."

We have thus endeavored to present to our readers an exposition of the views to which Dr. West has arrived in relation to the pathological value of ulceration of the os uteri and its proper treatment. We bespeak for his lectures a careful and candid study on the part of physicians of the United States, believing them to be calculated to impart important suggestions in reference to the true character and pathological relationship of these ulcerations, and their correct therapeutical management. The series of facts presented by the lecturer, and the deductions to which he believes them to lead, place ulceration of the neck and orifice of the womb in a point of view that has almost entirely escaped the attention of the leading writers on the subject, and indicate a plan of treatment of the cases in which it occurs more rational and less objectionable than the one now almost universally practised. D. F. C.

—*American Journal Medical Sciences.*

